# Prevalence, severity and correlates of dysmenorrhea among postpartum women in Western Kenya: A cross-sectional study

**DOI:** 10.1371/journal.pgph.0006466

**Published:** 2026-05-26

**Authors:** Madalitso Khwepeya, Jillian Pintye, Felix Abuna, Amritha Bhat, Daniel A. Enquobahrie, Lauren Gomez, Grace John-Stewart, John Kinuthia, Mary Marwa, Nancy Ngumbau, Ben Odhiambo, Salphine Watoyi, Joshua Stern, Anna Larsen

**Affiliations:** 1 Department of Epidemiology, School of Public Health, University of Washington, Seattle, Washington, United States of America; 2 Department of Behavioral Nursing and Health Informatics, School of Nursing, University of Washington, Seattle, Washington, United States of America; 3 Department of Global Health, University of Washington, Seattle, Washington, United States of America; 4 Department of Medical Research and Programs, Kenyatta National Hospital, Nairobi, Kenya; 5 Department of Psychiatry and Behavioral Sciences, School of Medicine, University of Washington, Seattle, Washington, United States of America; 6 Department of Medicine, University of Washington, Seattle, Washington, United States of America; 7 Department of Pediatrics, University of Washington, Seattle, Washington, United States of America; PLOS: Public Library of Science, UNITED STATES OF AMERICA

## Abstract

Dysmenorrhea is the most common menstrual disorder globally, contributing the highest disease burden of any gynecological issue in low- and middle-income countries (LMICs). Yet, limited data exist on dysmenorrhea among women in LMICs, particularly in Sub-Saharan Africa, including countries like Kenya. We conducted a cross-sectional analysis using data from the PrIMA-Extension Study, a large prospective cohort study that enrolled women during pregnancy and followed mother-child pairs up to 60 months post-delivery in four clinics in Western Kenya. At 24 months post-delivery, we assessed prevalence, severity, and correlates of dysmenorrhea using the Cox Menstrual Symptom Scale. Moderate-to-severe dysmenorrhea was defined as ≥1 symptom that was moderately to very severely bothersome. Poisson regression models identified correlates of moderate-to-severe dysmenorrhea. Among 277 women (median age: 27 years, IQR: 24–31), 24% (n = 67) had moderate-to-severe dysmenorrhea. Women predominantly reported cramps (46%), abdominal pain (34%), headaches (13%), weakness (8%), and dizziness (8%), lasting 3–7 hours or longer. Severe symptoms of dysmenorrhea most often reported were cramps (17%), abdominal pain (12%), headaches (4%), and weakness (3%). In a multivariate analysis adjusted for age and current breastfeeding status, moderate-to-severe dysmenorrhea was associated with high scores of adverse childhood experiences (ACEs; adjusted PR [aPR]=1.71, 95% CI: 1.04–2.79, p = 0.03), spending time in bed due to menstrual problems (aPR = 3.11, 95% CI: 1.88–5.14, p < 0.001), and taking painkillers (aPR = 4.12, 95% CI: 2.52–6.73, p < 0.001). Adjusting for age, current breastfeeding was associated with a higher frequency of moderate-to-severe dysmenorrhea (aPR = 1.66, 95% CI: 1.02–2.71, p = 0.04). At 2 years postpartum, nearly 1 in 4 women reported moderate-to-severe dysmenorrhea, associated with ACEs and resulting in daily functional interruptions. These findings underscore the need for routine menstrual health assessment during care and integrated approaches addressing pain management and psychosocial support.

## Introduction

Dysmenorrhea is the most prevalent menstrual disorder globally with the highest disease burden of any gynecological issue in low- and middle-income countries (LMICs) [1]. The global prevalence of dysmenorrhea ranges from 20% to 90% across different populations, depending on the method of assessment [2,3]. Approximately 10–15% of women experience dysmenorrhea severe enough to prevent normal daily functioning [1,2,4–7].

Dysmenorrhea presents as lower abdominal pain or cramping commonly accompanied by backache, nausea and vomiting, headache, dizziness, diarrhea, loss of appetite, bloating, leg pains, and irritability [8]. Dysmenorrhea symptoms especially when moderate to severe, often negatively impact women’s quality of life, work productivity, and healthcare utilization [9–12]. Yet, there are limited data on the frequency and severity of dysmenorrhea in LMICs, particularly in Sub-Saharan Africa (SSA) [9,12]—a region with persisting gaps in women’s health. Data on dysmenorrhea in this context could inform clinical and public health interventions and improve health and quality of life for those impacted.

Factors associated with dysmenorrhea include age, parity, marital status, menstrual bleeding characteristics, cigarette smoking, and alcohol consumption [9,10,12,13]. Hormonal contraceptive use may protect against dysmenorrhea symptoms [11,5]. Furthermore, experiences of dysmenorrhea are associated with anxiety, embarrassment, discomfort, and depression among women, especially younger women and adolescents [11]. Several studies demonstrate an increased risk of depression in women with dysmenorrhea, especially in the period after pregnancy, when postpartum depression is a health concern [14]. There is a dearth of data on dysmenorrhea among women in SSA [15]. Particularly, data are sparse regarding dysmenorrhea during the postpartum period in SSA, where high fertility rates in many countries mean many women spend a substantial proportion of their reproductive years in pregnant or postpartum states [16,17]. Understanding the burden and risk factors for dysmenorrhea up to 24 months post-delivery can inform postpartum health services to alleviate the negative impacts of dysmenorrhea and improve the quality of life.

We conducted a secondary analysis to determine the prevalence, severity, and correlates of moderate-to-severe dysmenorrhea at 24 months post-delivery, using data from a large cohort of women in Kenya. We hypothesized that the prevalence of moderate-to-severe dysmenorrhea would be high, and be associated with low parity, younger age, and irregular menstrual cycles, while hormonal contraceptive use would be inversely associated.

## Methods

### Study design and participants

We conducted a secondary cross-sectional analysis, leveraging data from women enrolled in the Pre-Exposure Prophylaxis (PrEP) Implementation for Mothers in Antenatal Care (PrIMA) Extension (PrIMA-X) Study (R01 HD100201), a longitudinal observational cohort study assessing the safety of PrEP during pregnancy and postpartum. PrIMA-X enrollment was conducted at four maternal and child health (MCH) clinics in Western Kenya between October 26, 2020, and June 6, 2023, and eligible participants were at least 15 years old, living without HIV, and were receiving MCH services at the study sites. Mother-child pairs were followed prospectively for up to 60 months postpartum.

In September 2022, data collection for dysmenorrhea symptoms was incorporated into study procedures. Data were included in the present analysis among women who had menstruation data recorded at their 24-month post-delivery visit from the time that dysmenorrhea measurement was activated through Dec 2, 2024. Among 1,191 women enrolled in PrIMA-X, 277 participants met the inclusion criteria for this analysis.

### Ethical consideration

Ethical approval was obtained from the University of Washington Institutional Review Board (STUDY00008710) and the Kenyatta National Hospital, University of Nairobi Ethics and Research Committee (KNH-UoN ERC: P921/11/2019). All participants provided written informed consent.

### Inclusivity in global research

Additional information regarding the ethical, cultural, and scientific considerations specific to inclusivity in global research is included in the Supporting Information (S1 Checklist).

### Data collection and measurements

Data were collected by trained study nurses using tablet-based Research Electronic Data Capture (REDCap) questionnaires [18]. Questionnaires were administered in English, Swahili, or Dholuo based on participant preference. All measures or variables used in this analysis were collected at various time points, including at 24 months post-delivery. They included items related to sociodemographic characteristics (age: ≥ 24 vs. 25–35 vs. > 35 years, marital status, employment, education level: ≥ 8 vs. < 8 years, etc.), obstetric information (pregnancy outcomes, breastfeeding, mode of delivery, non-permanent birth control methods, etc.), psychosocial characteristics (alcohol/tobacco, etc.) and menstrual patterns (length of menstrual cycle, bleeding days per period, nature of menstrual flow, hours spent on bed and/or pills taken in last menstrual period for menstrual relief).

#### Dysmenorrhea*.*

We used the Cox Menstrual Symptom Scale (CMSS) to measure duration and severity of physical and emotional dysmenorrhea symptoms [19]. The CMSS evaluates 18 symptoms of dysmenorrhea in terms of duration and severity, respectively, including general aching, headaches, stomachaches, backaches, cramps, leg aches, dizziness, facial blemishes, flushing, nausea, vomiting, loss of appetite, diarrhea, weakness, insomnia, gloominess, irritability, and nervousness, using a 5-point Likert scale. Duration is rated from 0 (“did not occur”) to 4 (“lasted several days”), and severity from 0 (“not noticeable”) to 4 (“very severely bothersome”). Item-level scores for duration and severity are summed to produce a total score ranging from 0 to 144, where higher scores indicate more severe symptoms [20]. Although the CMSS has not been formally validated in Swahili or Dholuo, the scale was translated and back-translated by bilingual experts and pilot-tested with local women to ensure conceptual clarity and linguistic appropriateness.

In the present analysis, the primary outcome was moderate-to-severe dysmenorrhea, defined as reporting at least one menstrual symptom that was moderately to very severely bothersome (CMSS severity score ≥2 vs. score <2).

#### Depressive symptoms*.*

Depressive symptoms were assessed using the 10-item Center for Epidemiologic Studies Depression Scale (CESD-10), a shortened version of the original CESD that has been validated in diverse populations, including adolescents and adults in SSA [21,22]. The CESD-10 includes 10 items rated on a 4-point Likert scale ranging from 0 (“rarely or none of the time”) to 3 (“most or all of the time”) based on symptom frequency in the past week. Total scores range from 0 to 30, with higher scores indicating greater severity of depressive symptoms. Consistent with prior research, scores were dichotomized as low (<10) and high (≥10) depressive symptoms to identify clinically meaningful distress [23,24].

#### Perceived stress*.*

Perceived stress was measured using the 10-item Perceived Stress Scale (PSS-10), a widely used psychological instrument designed to measure the degree to which individuals perceive life situations as stressful during the past month, on a 5-point Likert scale ranging from 0 (“never”) to 4 (“very often”) [25]. Four positively worded items were reverse-scored, and total scores ranged from 0 to 40, with higher scores indicating greater levels of perceived stress. For this analysis, perceived stress was dichotomized as low (<10) and high (≥10), with the threshold of 10 reflecting elevated stress in population-based studies [26].

#### Adverse childhood experiences (ACEs)*.*

ACEs were measured using the 31-item of Adverse Childhood Experiences International Questionnaire (ACE-IQ), capturing exposure to a range of adversities during childhood and adolescence, including physical, emotional, and sexual abuse; neglect; household dysfunction (e.g., caregiver substance use, incarceration, mental illness, death, or separation); bullying; and exposure to violence in the home, school, or broader community [27,28]. These items were grouped into 13 domains based on established ACE frameworks, with each domain coded as either present or absent. A cumulative ACE score (range: 0–13) was then calculated by summing the number of domains experienced. For analysis, ACE exposure was dichotomized as high (≥6) and low (<6), thresholds supported by evidence linking higher ACE burden to increased risk of adverse mental, behavioral, and physical health outcomes [28,29].

#### Intimate partner violence (IPV).

IPV was assessed using the Hurt, Insult, Threaten, and Scream (HITS) scale, a validated 4-item screening tool designed to measure the frequency of physical and emotional abuse by a partner [30]. The scale includes the items: Hurt, Insult, Threaten, and Scream, each rated on a 5-point Likert scale ranging from 1 (“never”) to 5 (“frequently”), yielding a total score range of 4–20. Higher scores indicate greater IPV exposure. We defined IPV based on thresholds established for identifying individuals at risk of IPV in clinical and research settings (HITS score ≥10 vs. < 10) [30].

#### Mother-child engagement.

Mother-child engagement was measured using items from the Multiple Indicator Cluster Survey (MICS) Early Childhood Development module, which assesses the frequency and quality of developmental stimulation activities provided by caregivers [31]. Mothers were asked whether they engaged in key activities with their child in the past three days, such as reading, storytelling, singing songs, playing, or naming/counting objects. Responses were used to create a binary variable indicating high engagement (if the mother participated in at least four of the six recommended activities) and low engagement (if fewer than four activities were reported), consistent with global early childhood development benchmarks [31].

### Data analysis

Descriptive statistics were used to summarize symptom duration and severity. Poisson regression with robust variance estimation, clustered by facility level, was used to assess the associations between moderate-to-severe dysmenorrhea and potential correlates. For the adjusted analyses, we selected age (as a continuous variable) and current breastfeeding status (Yes/No) as a priori confounders based on previous literature and a conceptual model of dysmenorrhea etiology [5]. Each variable with p ≤ 0.05 in univariate analysis was then entered into a separate multivariable Poisson model controlling for these covariates. R software (version 4.3.1, 2023-06-16) was used for analysis.

## Results

### Participant characteristics

**Fig 1:**
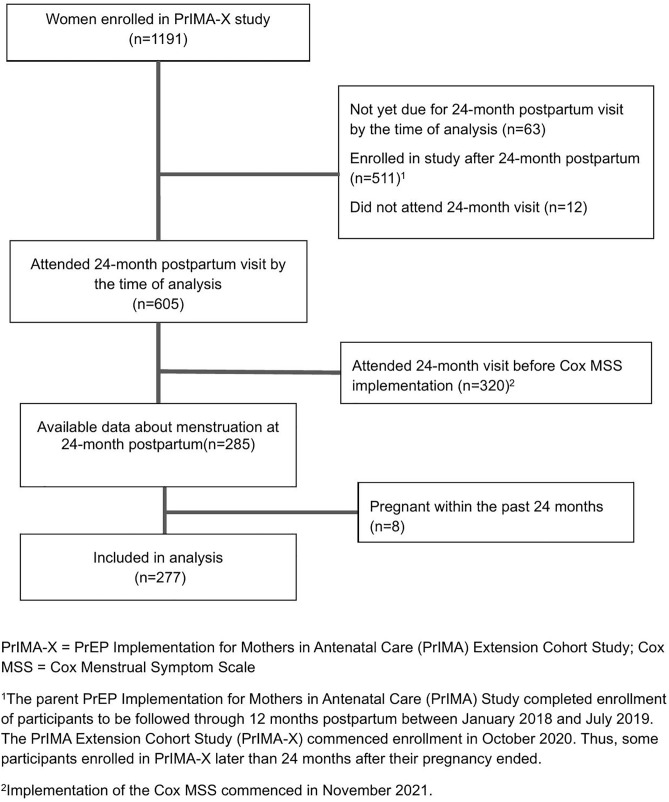
Flow diagram of enrollment into the study and participant inclusion in the current analyses.

A total of 277 women met the inclusion criteria and were included in the final analysis (Fig 1). The participants had a median age of 27 years [interquartile range (IQR) of 24–31, absolute range: 15–41, and most were aged between 25–35 years (59%)]. The majority were married or living with a partner (94%), in monogamous relationships (82%), and had completed more than eight years of education (77%). Nearly all had more than one child (93%) and reported a vaginal delivery (94%) for their most recent birth. At the time of data collection, nearly half were still breastfeeding (44%) and 97% were on contraception; the most commonly reported contraceptive methods were injectables (32%), followed by implants (28%) and other non-permanent methods (e.g., condoms; 26%).

Psychosocial distress factors were relatively rare. Only 2% of women reported depressive symptoms, 4% reported elevated stress, and less than 1% reported IPV. However, 27% reported exposure to six or more ACEs, and 16% had lower levels of mother-infant engagement. A full description of participant characteristics is provided in Table 1.

**Table 1:** Characteristics of participants included in the analysis (n = 277).

Demographic characteristics	N	n (%) or Median (IQR)
Age (years)	277	27 (24–31)
Age category (years)	277	
≥ 24		90 (32.49)
25-35		163 (58.85)
> 35		24 (8.66)
Marital status	277	
Single/divorced/widowed		16 (5.78)
Married/living with a partner		261 (94.22)
Type of marriage^1^	260	
Monogamous		213 (81.92)
Polygamous		47 (18.08)
Currently in school	277	
Yes		13 (4.69)
Number of complete years in school (years)	277	9 (8–12)
≥ 8		213 (76.90)
< 8		64 (23.10)
Regular employment^2^	276	
Yes		33 (11.96)
Currently taking PrEP	277	
Yes		188 (67.87)
HIV status	277	
Negative		274 (98.92)
Positive		3 (1.08)
**Pregnancy and clinical characteristics**		
Time since last pregnancy end (months)	277	24 (24.0-24.2)
Number of total pregnancies^3^	276	3 (2–4)
Number of total live births^3^	255	2 (1–3)
Number of livings children^3^	256	2 (1–3)
Outcome of most recent pregnancy	277	
Live birth		271 (97.83)
Spontaneous abortion		2 (0.72)
Stillbirth		4 (1.44)
Currently breastfeeding	269	
Yes		118 (43.87)
Mode of most recent delivery	214	
Vaginal		200 (93.46)
C-section		14 (6.54)
Use of non-permanent birth control^4^	270	
OCP		14 (5.05)
Injectable		88 (31.77)
IUD		5 (1.81)
Implants		77 (27.80)
Condoms		10 (3.61)
LAM		3 (1.08)
Other		73 (26.35)
**Psychosocial characteristics** ^**5**^		
Moderate to severe depression^6^	277	5 (3–6)
Yes		7 (2.53)
Moderate to severe perceived stress^7^	277	10 (8–11)
Yes		12 (4.33)
Adverse childhood experiences (ACEs)^8^	277	
High		75 (27.08)
Low		202 (72.92)
Alcohol and substance use^9^	277	
Never		271 (97.83)
Monthly or less		4 (1.44)
≥ 2/week		2 (0.72)
Intimate partner violence^10^	277	
Yes		3 (1.08)
Mother-infant engagement^11^	271	
High		226 (81.59)
Low		45 (16.25)
**Menstrual characteristics**		
Total dysmenorrhea score	277	2 (0-6)
Dysmenorrhea frequency symptom score	277	1 (0-4)
Dysmenorrhea severity symptom score	277	1 (0-3)
Moderate-to-severe dysmenorrhea^12^	277	
Yes		67 (24.19)
Length of menstrual cycle^13^	277	
Regular (20–40 days)		141 (50.90)
Irregular		136 (49.10)
Bleeding days per period^13^	277	
< 3 days		74 (26.71)
3–5 days		188 (67.87)
>=7 days		15 (5.42)
Nature of menstrual flow^13^	277	
Light		77 (27.80)
Moderate		173 (62.45)
Heavy		27 (9.75)
Any additional time spent on bed due to menstrual problems^14^	277	
Yes		58 (20.94)
Any pills taken in last menstrual period for menstrual relief^15^	277	
Yes		49 (17.69)

PrEP = Pre-exposure prophylaxis for HIV prevention; OCP = Oral contraceptive pills; IUD = Intrauterine device; LAM = Lactational amenorrhea method;

^1^Polygamous marriage was evaluated among those who are married.

^2^Regular employment was evaluated as participants reported engaging in an economic activity to earn a living wage.

^3^Pregnancy and obstetric history assessed among those with a history of prior pregnancy at enrollment.

^4^Non-permanent contraceptive type used was evaluated among those reporting currently using contraceptives; Other = None, abstinence, periodic abstinence, withdrawal, and emergency contraception.

^5^Assessed at 24 months post-delivery.

^6^Depression symptoms were assessed using the CESD and dichotomized as low (depression <10) and high (depression score ≥10).

^7^Stress symptoms were assessed using the perceived stress scale and dichotomized as low (pss < 14) and high (pss score ≥14).

^8^Adverse Childhood Experiences (ACEs) were assessed using the ACEs questionnaire and dichotomized as low (ACE score <6) and high (ACE score ≥6).

^9^Alcohol use was defined as consuming a drink containing alcohol at least once in the past month.

^10^Intimate Partner Violence using the HITS scale and dichotomized as low (negative score<10) and high (positive score ≥10).

^11^Mother and child engagement using the Multiple Indicator Cluster Survey (MICS) scale and dichotomized as Yes/No for High, and Low.

^12^Moderate-to-severe dysmenorrhea was defined as reporting at least one menstrual symptom that was moderately to very severely bothersome on the CMSS; dichotomized into Yes/No.

^13^Menstrual experiences in the last 6 months among those who reported menstruation.

^14^Additional time spent in bed was defined as extra bed rest by those experiencing menstrual problems throughout the last period; dichotomized as Yes (>0 hours)/No (0 hours).

^15^Pills taken for menstrual relief were evaluated among those who reported taking any kind of pills for menstrual relief over the number of days of medication (Yes/No).

### Dysmenorrhea symptoms

Overall, the median dysmenorrhea total score was 2 (IQR: 0–6), with 24% (n = 67) of mothers reporting moderate-to-severe dysmenorrhea symptoms defined as having at least one moderate to very severely bothersome symptom that lasted at least one full day on the CMSS. Over half of the women reported regular cycles (51%), and most had menstrual bleeding lasting 3–5 days (68%). The majority reported moderate flow levels (63%), while a smaller proportion spent additional time in bed (21%) or used painkillers to manage menstrual symptoms (18%) (Table 1).

Among those reporting any experience of individual dysmenorrhea symptoms, the most commonly experienced dysmenorrhea symptoms were cramps (46%), followed by abdominal pains (34%), headaches (13%), weakness (8%), and dizziness (8%), that varied in duration and typically lasted 3–7 hours or longer, with approximately 19% (n = 44) reporting symptoms that persisted for an entire day or more (Table 2). Among women with severe dysmenorrhea, 17% of women reported cramps, followed by abdominal pain (12%), headaches (4%), and weakness (3%) (Table 3).

**Table 2:** Duration of symptoms related to dysmenorrhea at 24 months since pregnancy end date (n = 277).

		Any experience of symptom^1^		Duration of symptom occurrence^2^				
	N	No	Yes	Did not occur	Lasted < 3 hours	Lasted 3–7 hours	Lasted entire day	Last several days
***Aches and pains***								
Cramps	277	151 (54.51%)	126 (45.49%)	151 (54.51%)	69 (24.91%)	30 (10.83%)	15 (5.42%)	12 (4.33%)
Headaches	274	239 (86.28%)	35 (12.64%)	239 (86.28%)	20 (7.22%)	11 (3.97%)	4 (1.44%)	–
Abdominal pain	276	182 (65.70%)	94 (33.94%)	182 (65.70%)	53 (19.13%)	24 (8.66%)	15 (5.42%)	2 (0.72%)
Leg aches	277	271 (97.83%)	6 (2.17%)	271 (97.83%)	6 (2.17%)	–	–	–
Sleeplessness	275	274 (98.92%)	1 (0.36%)	274 (98.92%)	1 (0.36%)	–	–	–
***Gastrointestinal issues***								
Nausea	277	266 (96.03%)	11 (3.97%)	266 (96.03%)	9 (3.25%)	1 (0.36%)	1 (0.36%)	–
Vomiting	277	277 (100.00%)	–	277 (100.00%)	–	–	–	–
Loss of appetite	277	267 (96.39%)	10 (3.61%)	267 (96.39%)	6 (2.17%)	2 (0.72%)	2 (0.72%)	–
Diarrhea	275	275 (99.28%)	–	275 (99.28%)	–	–	–	–
***Other symptoms***								
Facial blemishes	272	264 (95.31%)	8 (2.89%)	264 (95.31%)	6 (2.17%)	–	–	2 (0.72%)
Flushing	274	269 (97.11%)	5 (1.81%)	269 (97.11%)	5 (1.81%)	–	–	–
Dizziness	277	256 (92.42%)	21 (7.58%)	256 (92.42%)	15 (5.42%)	4 (1.44%)	2 (0.72%)	–
Irritability	274	265 (95.67%)	9 (3.25%)	265 (95.67%)	6 (2.16%)	2 (0.72%)	1 (0.36%)	–
Depression	275	273 (98.56%)	2 (0.72%)	273 (98.56%)	1 (0.36%)	–	1 (0.36%)	–
Nervousness	274	265 (95.67%)	9 (3.25%)	265 (95.67%)	9 (3.25%)	–	–	–
Weakness	277	254 (91.70%)	23 (8.30%)	254 (91.70%)	17 (6.13%)	5 (1.81%)	1 (0.36%)	–

^1^Experience of dysmenorrhea symptoms assessed among those reporting menstrual bleeding since the most recent pregnancy ended using the Cox MSS; dichotomized as “any experience of symptom”, Yes/No

^2^Duration of symptom occurrence evaluated among those who reported experiencing any of the symptoms based on the total amount of time one experienced a condition during her last period

**Table 3:** Severity of symptoms related to dysmenorrhea at 24 months since pregnancy end date (n = 277).

		Experience of moderate to severe symptoms		Severity of symptoms^1^				
	N	No	Yes	Not noticeable	Slightly bothersome	Moderate bothersome	Severely bothersome	Very severely bothersome
***Aches and pains***								
Cramps	272	224 (80.87%)	48 (17.33%)	150 (54.15%)	74 (26.71%)	42 (15.16%)	6 (2.17%)	–
Headaches	275	265 (95.67%)	10 (3.61%)	237 (85.55%)	28 (10.11%)	10 (3.61%)	–	–
Abdominal pain	277	243 (87.73%)	34 (12.27%)	187 (67.51%)	56 (20.22%)	28 (10.11%)	6 (2.17%)	–
Leg aches	275	275 (99.28%)	–	269 (97.11%)	6 (2.17%)	–	–	–
Sleeplessness	273	273 (98.56%)	–	271 (97.83%)	2 (0.72%)	–	–	–
***Gastrointestinal issues***								
Nausea	275	275 (99.28%)	–	265 (95.67%)	10 (3.61%)	–	–	–
Vomiting	276	276 (99.64%)	–	275 (99.28%)	1 (0.36%)	–	–	–
Loss of appetite	276	272 (98.20%)	4 (1.44%)	265 (95.67%)	7 (2.53%)	4 (1.44%)	–	–
Diarrhea	276	276 (99.64%)	–	275 (99.28%)	1 (0.36%)	–	–	–
***Other symptoms***								
Facial blemishes	277	276 (99.64%)	1 (0.36%)	270 (97.47%)	6 (2.17%)	1 (0.36%)	–	–
Flushing	276	276 (99.64%)	–	272 (98.20%)	4 (1.44%)	–	–	–
Dizziness	276	271 (97.83%)	5 (1.81%)	259 (93.50%)	12 (4.33%)	4 (1.44%)	1 (0.36%)	–
Irritability	276	273 (98.56%)	3 (1.08%)	266 (96.03%)	7 (2.53%)	3 (1.08%)	–	–
Depression	276	276 (99.64%)	–	276 (99.64%)	–	–	–	–
Nervousness	274	274 (98.92%)	–	267 (96.39%)	7 (2.53%)	–	–	–
Weakness	274	267 (96.39%)	7 (2.53%)	252 (90.98%)	15 (5.42%)	7 (2.53%)	–	–

^1^Severity of symptom occurrence evaluated using the Cox MSS among those who reported experiencing any of the symptoms based on the average level of pain or distress of the condition when it did occur in the last menstrual period

### Correlates of dysmenorrhea symptoms

In the univariate analysis, several factors were significantly associated with moderate-to-severe dysmenorrhea (Table 4). Women who reported higher scores of ACEs were more likely to have moderate-to-high dysmenorrhea than those with fewer ACEs (36.0% vs. 19.8%, prevalence ratio [PR]=1.82, 95% confidence interval [CI]: 1.10–2.95, p = 0.02). Women who were still breastfeeding at 24 months post-delivery had a higher risk of moderate-to-severe dysmenorrhea compared to those not breastfeeding (32.2% vs. 19.2%, PR = 1.68, 95% CI: 1.04–2.74, p = 0.04). Likewise, each unit increase in depressive symptom score was associated with a higher risk of moderate-to-severe dysmenorrhea (PR = 1.10, 95% CI: 1.01–1.18, p = 0.02). Moderate-to-severe dysmenorrhea was also related to pain relief behaviors: women with moderate-to-severe symptoms were more likely to use painkillers (67.3% vs. 14.9%, PR = 4.52, 95% CI: 2.79–7.30, p < 0.001) and spend additional hours in bed (55.2% vs. 16.0%, PR = 3.45, 95% CI: 2.13–5.58, p < 0.001) for symptom relief compared to those with less severe dysmenorrhea.

**Table 4:** Correlates of moderate to severe dysmenorrhea at 24 months post-delivery (n = 277).

		*n (%)*		*Univariate Poisson model*		*Multivariate Poisson model*	
	*N*	*Moderate-to-severe dysmenrrohea* ^*1*^		*PR (95% CI)*	*p-value* ^*1*^	*Adj PR (95% CI)*	*p-value* ^*1*^
Demographic characteristics		*No (N = 210)*	*Yes (N = 67)*				
Age category (years)	277						
≤ 24	90	72 (80.00)	18 (20.00)	0.74 (0.42–1.26)	0.284		
25-35	163	119 (73.01)	44 (26.99)	Ref			
> 35	24	19 (79.17)	5 (20.83)	0.77 (0.27–1.77)	0.583		
Married	277						
No	16	13 (81.25)	3 (18.75)	Ref			
Yes	261	197 (75.48)	64 (24.52)	1.31 (0.49–5.35)	0.650		
Monogamous	260						
No	47	35 (74.47)	12 (25.53)	Ref			
Yes	213	161 (75.59)	52 (24.41)	0.96 (0.53–1.88)	0.889		
Currently in school	277						
No	264	199 (75.38)	65 (24.62)	Ref			
Yes	13	11 (84.62)	2 (15.38)	0.62 (0.10–1.99)	0.512		
≥8 years school completion	277						
No	64	51 (79.69)	13 (20.31)	Ref			
Yes	213	159 (74.65)	54 (25.35)	1.25 (0.70–2.39)	0.473		
Regular employment	276						
No	243	184 (75.72)	59 (24.28)	Ref			
Yes	33	25 (75.76)	8 (24.24)	1.00 (0.44–1.97)	0.997		
**Pregnancy and clinical characteristics**							
Pregnancy loss	277						
No	271	205 (75.65)	66 (24.35)	Ref			
Yes	6	5 (83.33)	1 (16.67)	0.68 (0.04–3.09)	0.707		
Currently breastfeeding	269						
No	151	122 (80.79)	29 (19.21)	Ref		*Ref	
Yes	118	80 (67.80)	29 (32.20)	1.68 (1.04–2.74)	0.036	1.66 (1.02–2.71)	0.041
Mode of delivery	214						
C-section	14	13 (92.86)	1 (7.14)	Ref			
Vaginal	200	154 (77.00)	46 (23.00)	3.22 (0.71–57.04)	0.247		
Non-permanent birth controls	270						
Other	91	70 (76.92)	21 (23.08)	Ref			
OCP	14	11 (78.57)	3 (21.43)	0.93 (0.22–2.69)	0.904		
Injectable/Implants	165	124 (75.15)	41 (24.85)	1.08 (0.64–1.86)	0.783		
**Psychosocial characteristics**							
Depressive symptoms score	277			1.10 (1.01–1.18)	0.016	**1.08 (0.9–1.18)	0.068
Moderate to severe depression							
No	270	207 (76.67)	63 (23.33)	Ref			
Yes	7	3 (42.86)	4 (57.14)	2.45 (0.74–5.93)	0.082		
Perceived stress score	277			0.96 (0.87–1.05)	0.407		
Moderate to severe perceived stress							
No	265	202 (76.23)	63 (23.77)	Ref			
Yes	12	8 (66.67)	4 (33.33)	1.40 (0.43–3.40)	0.512		
High ACEs	277						
No	202	162 (80.20)	40 (19.80)	Ref		**Ref	
Yes	75	48 (64.00)	27 (36.00)	1.82 (1.10–2.95)	0.016	1.71 (1.04–2.79)	0.032
Intimate partner violence	277						
No	274	209 (76.28)	65 (23.72)	Ref			
Yes	3	1 (33.33)	2 (66.67)	2.81 (0.46 – 8.96)	0.150		
Mother-infant engagement	271						
Low	45	39 (86.67)	6 (13.33)	Ref			
High	226	166 (73.45)	60 (26.55)	1.99 (0.93–5.16)	0.108		
**Menstrual characteristics**							
Irregular menstrual cycle	277						
No	141	114 (80.85)	27 (19.15)	Ref			
Yes	136	96 (70.59)	40 (29.41)	1.54 (0.95–2.53)	0.085		
Bleeding days	277						
< 3 days	74	60 (81.08)	14 (18.92)	Ref			
3–5 days	188	136 (72.34)	52 (27.66)	1.46 (0.83–2.74)	0.207		
>=7 days	15	14 (93.33)	1 (6.67)	0.35 (0.02–1.75)	0.313		
Menstrual flow	277						
Light	77	58 (75.32)	19 (24.68)	Ref			
Moderate	173	133 (76.88)	40 (23.12)	0.94 (0.55–1.65)	0.815		
Heavy	27	19 (70.37)	8 (29.63)	1.20 (0.50–2.65)	0.664		
Additional time spent on bed due to menstrual problems	277						
No	219	184 (84.02)	35 (15.98)	Ref		**Ref	
Yes	58	26 (44.83)	32 (55.17)	3.45 (2.13–5.58)	<0.001	3.11 (1.88–5.14)	<0.001
Any pills taken in last menstrual period for menstrual relief	277						
No	228	194 (85.09)	34 (14.91)	Ref		**Ref	
Yes	49	16 (32.65)	33 (67.35)	4.52 (2.79–7.30)	<0.001	4.12 (2.52–6.73)	<0.001

PR=Prevalence ratio; CI=Confidence interval.

^1^Moderate-to-severe dysmenorrhea was defined as reporting at least one menstrual symptom that was moderately to very severely bothersome; dichotomized into Yes/No.

*Adjusted for age (continuous).

**Adjusted for age (continuous) and breastfeeding.

Although age, irregular menstrual cycles, and hormonal contraceptives were not significantly associated with having moderate-to-severe dysmenorrhea as hypothesized, they demonstrated trends in the expected direction, warranting further investigation. Being ≤24 years (20.0%, PR = 0.74, 95% CI: 0.42–1.26, p = 0.28) or >35 years (20.8%, PR = 0.77, 95% CI: 0.27–1.77, p = 0.58) compared to 25–35 years (27.0%) was associated with a lower risk of moderate-to-severe dysmenorrhea. Similarly, using oral contraceptive pills compared to other methods such as abstinence or periodic abstinence was associated with a slightly lower risk of moderate-to-severe dysmenorrhea (21.4% vs. 23.1%, PR = 0.93, 95% CI: 0.22–2.69, p = 0.90), while injectable or implants use showed a slightly higher risk of moderate-to-severe dysmenorrhea (24.8% vs. 23.1%, PR = 1.08, 95% CI: 0.64–1.86, p = 0.78). Irregular menses posed a greater risk of moderate-to-severe dysmenorrhea than regular menses (29.4% vs. 19.1%, PR = 1.54, 95% CI: 0.95–2.53, p = 0.09).

In multivariate analyses, several associations remained statistically significant after adjusting for maternal age and current breastfeeding. Participants who reported higher ACEs more frequently experience moderate-to-severe dysmenorrhea than those with fewer ACEs (adjusted PR [aPR]=1.71, 95% CI: 1.04–2.79, p = 0.03). Women who were breastfeeding at 24 months post-delivery had a higher likelihood of reporting moderate-to-severe dysmenorrhea compared to those who were not breastfeeding (aPR = 1.66, 95% CI: 1.02–2.71, p = 0.04), after adjustment for age. Pain relief behaviors were also significantly associated with severity: women with moderate-to-severe dysmenorrhea reported using painkillers more frequently (aPR = 4.12, 95% CI: 2.52–6.73, p < 0.001) and spending additional hours in bed than those without moderate-to-severe dysmenorrhea (aPR = 3.11, 95% CI: 1.88–5.14, p < 0.001).

## Discussion

This study aimed to assess the prevalence and severity of dysmenorrhea and identify its correlates among women at 2 years postpartum in Western Kenya. We found that 24% of women reported moderate-to-severe dysmenorrhea on the severity subscale of the CMSS, defined as experiencing at least one symptom that was moderately to very severely bothersome. Consistent with our hypothesis, abdominal cramps and pain were the most commonly reported symptoms, both in terms of duration and severity. These findings highlight a significant symptom burden among postpartum women in this setting, which is often underrecognized in both clinical and research contexts.

The overall prevalence of moderate-to-severe dysmenorrhea in our study was higher than previously reported estimates in LMICs, which typically range from 10% to 15% for dysmenorrhea severe enough to disrupt daily functioning, particularly among adolescents and young adult’s women, using a variety of symptom-based scales [1,2,4–7]. Our focus on a postpartum population with previous pregnancies and current caregiving responsibilities may account for this discrepancy. While parity is often associated with reduced risk of dysmenorrhea [5], other factors such as postpartum hormonal changes, uterine recovery, and caregiving demands may influence the menstrual pain trajectories. After childbirth, the uterus undergoes involution and physiological adaptation, including reduced prostaglandin production and decreased nerve fiber density, which can lower uterine contractility and pain sensitivity during menstruation [32]. Additionally, cultural normalization of menstrual pain and underreporting in face-to-face surveys may have contributed to the lower estimates elsewhere [15]. These findings highlight the complex biopsychosocial dimensions of menstrual pain in postpartum populations and point to the need for integrated postpartum care that considers trauma, caregiving demands and cultural norms surrounding help seeking and pain management.

Our finding that high ACEs were associated with moderate-to-severe dysmenorrhea in our study aligns with a growing body of evidence that early life adversity increases pain sensitivity and risk for chronic pain conditions, including menstrual disorders [33–35]. ACEs have been shown to dysregulate the hypothalamic-pituitary-adrenal (HPA) axis, heighten systemic inflammation, and alter pain processing pathways, all of which may predispose women to gynecological pain syndromes later in life [33,36,37]. Systematic reviews and cohort studies indicate that childhood exposure to violence, neglect, or household dysfunction is associated with both primary and secondary dysmenorrhea, as well as pelvic pain and endometriosis [34,35]. Specifically, women with a history of sexual abuse or emotional trauma are more likely to report severe menstrual pain, even after adjusting for sociodemographic factors [34,35]. These findings support a biopsychosocial framework for dysmenorrhea, emphasizing that menstrual pain is shaped not only by physiological and hormonal processes but also by the long-term psychological and neurobiological consequences of early trauma [33,38]. Integrating trauma-informed approaches into menstrual health care may be essential for addressing the needs of women with high ACE exposure.

The association between breastfeeding and moderate-to-severe dysmenorrhea observed in our study adds to a limited and conflicting body of evidence. Some research suggests that prolonged breastfeeding may delay the return of regular ovulatory cycles, potentially modulating ovarian hormone production and uterine activity, thereby offering temporary protection against menstrual pain [39–41]. For instance, a prospective observational study found that breastfeeding, particularly if exclusive, may cause improvement in dysmenorrhea and chronic pelvic pain proportional to the duration of breastfeeding, as well as a reduction in the size of ovarian endometriomas [42]. However, our finding that extended breastfeeding was associated with increased likelihood of moderate-to-severe dysmenorrhea may reflect the hormonal fluctuations and uterine involution that occur during lactation, particularly in the transition to regular menses [43]. Oxytocin-induced uterine contractions during breastfeeding accelerate involution but can also cause postpartum afterpains, which may intensify discomfort as regular menstruation resumes [43]. Additionally, the physical and emotional stress associated with caregiving and lactation demands might exacerbate pain perception in some women [44–46]. These findings suggest that the relationship between breastfeeding and dysmenorrhea is likely mediated by complex physiological and psychosocial mechanisms, underscoring the importance of considering postpartum status in menstrual health research.

Moderate-to-severe dysmenorrhea was associated with spending additional time in bed or taking painkillers during menstruation. These behaviors may reflect greater symptom intensity and also may indicate reliance on passive coping strategies that could unintentionally reinforce pain-related interruptions to functioning. Prolonged rest or withdrawal from daily activities has been associated with heightened pain sensitivity and reduced psychological resilience in individuals with chronic pelvic pain [47,48]. These findings emphasize the importance of equipping women with proactive, evidence-based pain management strategies and addressing structural barriers to care that may limit access to treatments, including trauma histories and caregiving demand.

We found that moderate-to-severe dysmenorrhea was associated with depression in univariate analysis. While this association was no longer significant after adjusting for age and breastfeeding, the point estimate remained high. Our findings are consistent with previous research indicating a strong co-occurrence between menstrual pain and depressive symptoms, potentially influenced by shared neuroendocrine, inflammatory, and psychological mechanisms [49–51]. Evidence also suggests that dysmenorrhea is associated with an increased risk of postpartum depression, highlighting a potential bidirectional relationship between pain and mood [14]. Meta-analyses and genetic studies further indicate that women with primary dysmenorrhea are more likely to experience depressive symptoms, and that depression may be linked to an increased risk of dysmenorrhea through pathways involving sleep disturbances and serotonin regulation [52]. Our results reinforce the importance of screening for mood symptoms in dysmenorrhea care. Further studies that are specifically designed to evaluate associations between mood and dysmenorrhea are needed to better understand the nature and strength of this relationship, particularly among women in the extended postnatal period.

Contrary to our secondary hypotheses, moderate-to-severe dysmenorrhea was not significantly associated with younger age, low parity, or irregular menstrual cycles, factors that have been associated with higher dysmenorrhea risk in previous studies among adolescents and nulliparous women [5,53,54]. The lack of association in our study may reflect the postpartum status of participants, who were generally older, multiparous, and currently breastfeeding. Pregnancy and childbirth may physiologically reduce menstrual pain through mechanisms such as hormonal reset or uterine recovery [5,55]. Multiparity was found to be protective against dysmenorrhea in prior studies, likely due to long-term changes in uterine innervation, reduced prostaglandin sensitivity, and anatomical changes that follow repeated pregnancies [5]. Additionally, since all participants had given birth, our sample may underrepresent individuals with conditions such as endometriosis, chronic pelvic inflammatory disease, or other conditions associated with subfertility, which are often associated with severe dysmenorrhea [56–58]. This selection bias may partially account for the absence of associations seen in broader populations [59].

Our study is among the first to provide epidemiological data from a large, well-characterized cohort on the prevalence, severity, and correlates of dysmenorrhea among postpartum women in Kenya, addressing a significant gap in this region. However, the study is not without limitations, which should be considered when interpreting results. First, the findings are based on a sample enrolled from two counties in Kenya, which may limit generalizability to other regions. However, over 90% of pregnant women in these counties attend at least one antenatal care visit; thus, enrolling in antenatal care clinics is likely to result in a representative sample of pregnant women [60]. Second, our study only included women who were pregnant within the past 24 months; therefore, our findings are not generalizable to women without prior pregnancies and those with conditions that may prevent successful pregnancies (e.g., infertility). Pre-pregnancy dysmenorrhea status was not assessed, and pregnancy and childbirth may alter menstrual pain through hormonal and physiological changes, limiting our ability to assess changes in symptom severity across reproductive stages. Furthermore, selection bias maybe possible if women with fewer symptoms or lower healthcare needs were less likely to return for follow-up. Third, although menstrual flow characteristics (light, moderate, or heavy) were collected, they were not significantly associated with moderate-to-severe dysmenorrhea in the univariate analysis and thus, was not included in the multivariate model; nevertheless, heavy menstrual bleeding may still contribute to dysmenorrhea severity in other populations. Fourth, the assessment and determination of dysmenorrhea relied on self-report questionnaires rather than clinical diagnosis, meaning we were unable to fully distinguish primary from secondary dysmenorrhea, and diagnoses such as endometriosis, pelvic inflammatory disease, or other reproductive tract conditions may have been missed. In addition, data on postpartum infections (e.g., sepsis), sexually transmitted infections other than HIV, and other comorbidities that may influence menstrual pain were not collected. Fifth, while education level and employment status were included as proxy indicators of socioeconomic status, more comprehensive measures (e.g., income or household wealth) were not available and may influence health-seeking behaviors, pain management strategies, and psychological distress. Finally, the scarcity of local studies assessing the prevalence and correlates of dysmenorrhea in Kenya may hinder external validity and limit comparisons. Future research should incorporate approaches for clinical exclusion of underlying pathology when possible. Despite these limitations, this study contributes valuable epidemiological data to bridge existing gaps and highlights the need for future research to explore associations between menstrual health and psychological distress among perinatal African women, including the need for longitudinal, clinically informed studies that capture menstrual health across the reproductive life course.

## Conclusion

This study assessed the prevalence, severity, and correlates of dysmenorrhea among postpartum women in Western Kenya. Nearly 1 in 4 women in this cohort experienced moderate-to-severe dysmenorrhea, with abdominal cramps and pain being the most commonly reported symptoms. Moderate-to-severe dysmenorrhea was associated with psychosocial and behavioral factors, including extended breastfeeding, high scores of ACEs, and coping behaviors such as additional time spent in bed and use of painkillers. These results highlight the complex biopsychosocial dimensions of menstrual pain and underscore the need for targeted, context-specific interventions to address dysmenorrhea.

## Supporting information

S1 Checklist Inclusivity in global research checklist.
(DOCX)

S1 File Dataset used for analysis.
(CSV)
